# DiabeticSense: A Non-Invasive, Multi-Sensor, IoT-Based Pre-Diagnostic System for Diabetes Detection Using Breath

**DOI:** 10.3390/jcm12206439

**Published:** 2023-10-10

**Authors:** Ritu Kapur, Yashwant Kumar, Swati Sharma, Vedant Rastogi, Shivani Sharma, Vikrant Kanwar, Tarun Sharma, Arnav Bhavsar, Varun Dutt

**Affiliations:** 1 Indian Knowledge System and Mental Health Applications Centre, Indian Institute of Technology Mandi, Kamand 175075, Himachal Pradesh, India; ritu_khosla@projects.iitmandi.ac.in (R.K.); s22039@students.iitmandi.ac.in (Y.K.); swati_sharma@projects.iitmandi.ac.in (S.S.); b21027@students.iitmandi.ac.in (V.R.); s23063@students.iitmandi.ac.in (S.S.); arnav@iitmandi.ac.in (A.B.); 2 All India Institute of Medical Science Bilaspur, Noa 174001, Himachal Pradesh, India; drkanwarvikrant@gmail.com (V.K.); tarunpgi@gmail.com (T.S.)

**Keywords:** digital health devices, diabetes test, bio-markers, blood glucose monitoring, diabetes, exhaled breath analysis, non-invasive, volatile organic compounds

## Abstract

Diabetes mellitus is a widespread chronic metabolic disorder that requires regular blood glucose level surveillance. Current invasive techniques, such as finger-prick tests, often result in discomfort, leading to infrequent monitoring and potential health complications. The primary objective of this study was to design a novel, portable, non-invasive system for diabetes detection using breath samples, named DiabeticSense, an affordable digital health device for early detection, to encourage immediate intervention. The device employed electrochemical sensors to assess volatile organic compounds in breath samples, whose concentrations differed between diabetic and non-diabetic individuals. The system merged vital signs with sensor voltages obtained by processing breath sample data to predict diabetic conditions. Our research used clinical breath samples from 100 patients at a nationally recognized hospital to form the dataset. Data were then processed using a gradient boosting classifier model, and the performance was cross-validated. The proposed system attained a promising accuracy of 86.6%, indicating an improvement of 20.72% over an existing regression technique. The developed device introduces a non-invasive, cost-effective, and user-friendly solution for preliminary diabetes detection. This has the potential to increase patient adherence to regular monitoring.

## 1. Introduction

Type 2 diabetes mellitus (T2DM) is a chronic metabolic disorder with high blood sugar levels (hyperglycemia). This is caused by inadequate insulin production by the pancreas or the body’s inability to effectively use the insulin produced, a condition known as insulin resistance [1]. The International Diabetes Federation (IDF) and World Health Organization (WHO) report approximately 500 million T2DM cases worldwide and estimate it to rise to around 800 million by 2045 [2,3]. T2DM, if left untreated or ignored, can lead to serious health complications, including damage to the eyes, blood vessels, kidneys, nerves, heart, and feet. These complications can lead to long-term disability and premature death. According to the WHO, an estimated 1.6 million deaths were directly attributed to diabetes in 2019, making it the seventh leading cause of mortality worldwide. Moreover, the burden of undiagnosed cases (46.1%) remains alarmingly high, with many individuals being diagnosed only at advanced stages. In India, for instance, approximately 101 million people are already diagnosed with diabetes, and 136 million are in the pre-diabetic stages, highlighting the urgent need for early detection and intervention.

Electronic noses comprising electronic sensors capable of smell or odor detection have found their application in various areas such as food, wine, material, tea, environment, and healthcare. In particular, their ability to discern subtle variations in scent profiles has opened up exciting possibilities for improving diagnostics and disease monitoring. For example, research has shown that the concentration of certain volatile organic compounds (VOCs) differs significantly in diabetic individuals compared to non-diabetic individuals [4]. This is because VOCs are produced by the body as a byproduct of metabolism, and their levels can be affected by a variety of factors, including disease.

Electronic sensors that are sensitive to specific VOCs can be used to detect these changes in breath and potentially provide early warning of disease. Researchers have shown that certain biomarkers in the human body are related to the incidence of certain diseases [5,6]. For instance, methanol, ethanol, acetone, isoprene, isopropanol, propane, and undecane have been found to be associated with lung cancer [7], and acetone has been found to be associated with diabetes and heart failure [8]. A study showed that an electronic nose could be used to distinguish between diabetic and non-diabetic individuals with an accuracy of 90% [9]. This is a promising development, as current methods for diagnosing diabetes, such as finger-prick tests, can be invasive and uncomfortable for patients, leading to infrequent monitoring and potential health complications [10,11]. Electronic noses could provide a more non-invasive and convenient way to monitor blood sugar levels and detect diabetes early, which could lead to better health outcomes. Also, the existing non-invasive methods are mainly based on breath simulation data [12] and probabilistic estimation techniques [13]. To the best of our knowledge, there exist few (or none) non-invasive diabetes detection systems that are capable of detecting diabetes from breath using electronic sensors.

Therefore, to bridge the current gap in the literature, we propose a portable, non-invasive device for diabetes detection using breath, named DiabeticSense, incorporating cutting-edge metal–oxide–semiconductor (MOS)-type electrochemical sensors capable of detecting VOCs in a patient’s breath. Leveraging this unique biomarker pattern of a diabetic person, our non-invasive diabetes detection system offers an excellent pre-diagnostic tool for identifying diabetes at its early stage. The portability and affordability of our device make it particularly suitable for deployment in remote health centers, where access to comprehensive medical facilities may be limited. By providing a convenient and user-friendly alternative to traditional invasive tests, our non-invasive diabetes detection device empowers healthcare professionals in resource-constrained settings to perform timely and accurate diabetes screenings.

In this paper, we present the development and methodology of our non-invasive diabetes detection device, along with its promising performance in detecting diabetes. Additionally, we discuss the potential implications of this breakthrough technology in enhancing diabetes management and improving overall health outcomes, particularly in regions where early detection can significantly impact disease progression and reduce diabetes-related complications.

## 2. Background

The concept of non-invasive glucose monitoring has emerged as a central focus in diabetes care, with the aim of replacing or reducing reliance on traditional invasive methods. Among these alternatives, breath-based analysis, which detects VOCs indicative of glucose metabolism, has shown great promise. Advancements in machine learning (ML) algorithms have further strengthened this approach, enabling precise pattern recognition from complex breath samples.

The correlation between specific VOCs in human breath and blood glucose levels (BGLs) has been established. For instance, breath acetone has been identified as a crucial biomarker for type 2 diabetes, directly linked to blood glucose concentrations [14,15]. Previous studies have shown that the acetone concentration in breath correlates with BGLs [16]. This correlation has been used to develop non-invasive methods for detecting diabetes and monitoring BGLs [13]. Similarly, a recent study [17] investigated the potential of salivary amylase as a biomarker for diabetes. Salivary amylase studies have shown that the acetone concentration in breath correlates with BGLs. The authors validated that BGLs were significantly higher in patients with diabetics than in those without, highlighting the need for further research to validate these findings and develop accurate evaluation techniques. Understanding how a combination of VOCs in breath represents glucose levels remains a significant area of exploration.

The widespread adoption of smartphone technology has revolutionized diabetic care through digital interventions. Diabetes coaching applications have emerged to prevent complications and enhance self-management. A notable study [18] showcased the substantial benefits of a smartphone application in diabetes intervention. Similarly, the impact of smartphone applications on self-management among adults with type 1 diabetes was assessed, emphasizing their integral role in contemporary diabetes care [19]. However, both studies were limited by small sample sizes and short intervention durations.

Furthermore, sensor technology advancements, particularly electronic nose (E-Nose) systems, have significantly improved the specificity and sensitivity of VOC detection. These systems have found applications in various medical domains and even in identifying cigarette brands, illustrating their versatility and broad potential [4,13,20,21], thus highlighting the feasibility of non-invasive blood glucose monitoring through breath signal analysis. Nevertheless, rigorous testing of these systems for blood glucose monitoring via breath is still needed.

For instance, the authors in [12] presented the development of a human breath analysis system employing solid-state electrochemical sensors based on a digital nose framework. This study emphasized the need for an artificial-intelligence-driven predictive methodology due to the diverse range of breath components. However, efficient power management strategies for handling thermal aspects of the device for high-efficiency performance were yet to be explored for specific use cases. Similarly, the authors in [22] underscored the significance of power efficiency in output voltage regulation provided by switching regulators but raised concerns about undesirable ripples in switching regulators that might hinder the high-performance operation of embedded systems.

ML models have been applied to leverage breath data for diabetes detection, demonstrating a high degree of accuracy and showcasing the expansive potential of computational methods in medical diagnostics [12]. The versatility of these models is further demonstrated by their applicability to diverse datasets, as observed with the Chinese diabetes datasets [23]. The authors in [24] developed a predictive model to identify diabetes mellitus patients at risk of losing protective sensation in the foot. The model considered three factors: educational level, presence of neuropathic symptoms, and duration of diabetes. It successfully identified 81.3% of at-risk patients, suggesting its potential utility in helping patients take preventive measures to reduce their risk of developing foot ulcers and other complications.

However, despite these advancements, an observable gap remains. While there is growing research on breath-based glucose monitoring and ML for medical diagnostics, the literature remains sparse regarding synthesising a comprehensive array of sensors for breath sampling with auxiliary physiological parameters, such as blood pressure, age, gender, and SpO${}_{2}$ levels. Integrating these would potentially refine predictive accuracy, signifying an area yet to be extensively explored.

DiabeticSense proposed in this research overcomes several limitations in the existing literature. Specifically, it considers a plurality of VOCs, a device with a proper filter and power management, and ensemble ML algorithms for classifying diabetes.

## 3. Materials and Methods

### 3.1. Study Aim

To develop and evaluate a low-cost portable, non-invasive multi-sensor diabetes detection device (named DiabeticSense) that uses breath samples and body vitals as input and generates diabetes predictions based on ML models.

### 3.2. Design

**Figure 1:**
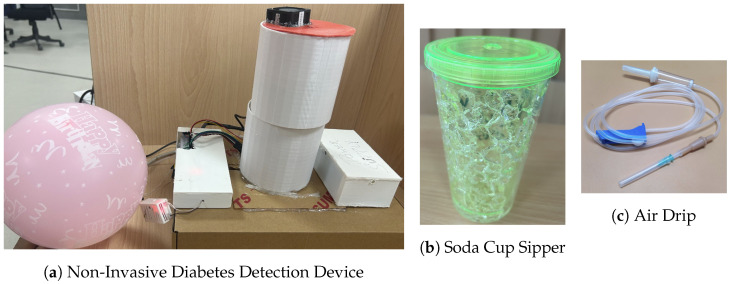
Breath sample collection and analysis device arrangement.

DiabeticSense (Figure 1a) comprises a sensor array of various MOS-type electrochemical sensors arranged in a cylindrical manner and integrated into a soda sipper cup by tightly closing the cap (Figure 1b). Birthday balloons were used to collect breath samples, and a drip pipe was used to slowly infuse the breath sample into the device (Figure 1c).

The sensors’ specifications are outlined in Table 1. These sensors were meticulously linked to a microcontroller via a 16-bit analog-to-digital converter (ADC), specifically the ADS1115. The interfacing between the microcontroller and the ADS1115 was facilitated using an I2C communication protocol. A WiFi-enabled ESP32 microcontroller was used in this experiment to facilitate network interaction. Communication between the microcontroller and external entities was effectuated by utilizing the MQTT protocol and establishing a link with a remote MQTT server within the network. The streaming of MQTT-derived data was channeled into an InfluxDB (https://www.influxdata.com/products/influxdb-cloud/, (accessed on 23 September 2023)) time series database. For a visualization and comprehensive examination of the device’s response, the InfluxDB was managed and visually represented using the Grafana (https://grafana.com/grafana/dashboards/ (accessed on 23 September 2023)). This enabled insightful analysis of the device’s performance.

### 3.3. Details of Sensors Used

To develop our multi-sensor breath analysis device, we used an array of sensors that include TGS 822, TGS 826, TGS 2600, TGS 2602, TGS 2603, TGS 2610, TGS 2620, and MQ 138. All the TGS sensors were manufactured by Figaro Inc., Osaka, Japan, while the MQ 138 sensor was manufactured by Zhengzhou Winsen Electronics Technology Co., Ltd., Zhengzhou, China. The sensors that we used have sensitivity to a broad range of gases, and are not sensitive to any one particular gas. Thus, our sensors are not highly specific to a particular gas. We also used DHT 22 sensor to record the temperature and humidity while conducting our experiments. DHT 22 is manufactured by the Aosong Electronics Co., Ltd., Guangzhou, China.

1. TGS 826: The sensing element of TGS 826 is a metal–oxide–semiconductor that has low conductivity in clean air. In the presence of a detectable gas, the sensor’s conductivity increases depending on the gas concentration in the air. A simple electrical circuit can convert the change in conductivity to an output signal corresponding to the gas concentration. The TGS 826 has sensitivity to VOCs such as iso-butane, ethanol, ammonia, and hydrogen gas. The sensor can detect concentrations as low as 30 ppm in the air and is ideally suited to critical safety-related applications such as the detection of ammonia leaks in refrigeration systems and ammonia detection in the agricultural field [25].
2. TGS 2610: TGS 2610 is a semiconductor-type gas sensor that combines very high sensitivity to Liquefied petroleum (LP) gas with low power consumption and long life. Due to the miniaturization of its sensing chip, TGS 2610 requires a heater current of only 56 mA and the device is housed in a standard TO-5 package. The TGS 2610 is available in two different models with different external housings but identical sensitivity to LP gas. Both models can satisfy the requirements of performance standards such as UL1484 and EN50194. TGS 2610-C00 possesses a small size and quick gas response, making it suitable for gas leakage checkers. TGS 2610-D00 uses filter material in its housing, eliminating the influence of interference gasses such as alcohol, resulting in a highly selective response to LP gas. This feature makes the sensor ideal for residential gas leakage detectors, which require durability and resistance against interference gas [26]. TGS 2610 shows sensitivity to ethanol, hydrogen, methane, iso-butane, and propane gas.
3. TGS 822: The sensing element of TGS 822 Figaro gas sensors is a tin dioxide (Sn$O_{2}$) semiconductor with low conductivity in clean air. In the presence of a detectable gas, the sensor’s conductivity increases depending on the gas concentration in the air. A simple electrical circuit can convert the change in conductivity to an output signal corresponding to the gas concentration. The TGS 822 is highly sensitive to the vapors of organic solvents and other volatile vapors. It is also sensitive to combustible gasses such as carbon monoxide, making it an excellent general-purpose sensor. It is also available with a ceramic base highly resistant to severe environments as high as 200 °C (in TGS 823). The complete list of gases that TGS 822 is sensitive to includes methane, carbon monoxide, iso-butane, n-hexane, benzene, ethanol, and acetone [27].
4. TGS 2602: The sensing element consists of a metal–oxide–semiconductor layer formed on the alumina substrate of a sensing chip together with an integrated heater. The TGS 2602 is highly sensitive to low concentrations of odorous gasses such as hydrogen, hydrogen sulfide, and ammonia generated from waste materials in office and home environments. The sensor is also susceptible to low concentrations of VOCs, such as toluene and ethanol emitted from wood finishing and construction products. Due to the miniaturization of the sensing chip, TGS 2602 requires a heater current of only 56 mA and the device is housed in a standard TO-5 package [28].
5. TGS 2600: This sensor is highly sensitive to low concentrations of gaseous air contaminants in cigarette smoke, such as hydrogen, methane, and carbon monoxide, and also shows sensitivity to iso-butane and ethanol. In the presence of a detectable gas, the sensor’s conductivity increases depending on the gas concentration in the air. The sensor can detect hydrogen at a level of several ppm. Due to the miniaturization of the sensing chip, TGS 2600 requires a heater current of only 42 mA and the device is housed in a standard TO-5 package [29].
6. TGS 2603: The sensing element consists of a metal–oxide–semiconductor layer formed on an alumina substrate of a sensing chip together with an integrated heater. In the presence of a detectable gas, the sensor’s conductivity increases depending on the gas concentration in the air. The TGS 2603 is highly sensitive to low concentrations of odorous gasses such as amine-series and sulfurous odor generated from waste materials or spoiled foods such as fish, such as methyl mercaptan and trimethyl amine, and is also sensitive to hydrogen sulfide, hydrogen, and ethanol. By utilising the change ratio of sensor resistance from the resistance in clean air as the relative response, human perception of air contaminants can be simulated and practical air quality control can be achieved [30].
7. TGS 2620: The sensing element consists of a metal–oxide–semiconductor layer formed on an alumina substrate of a sensing chip together with an integrated heater. In the presence of a detectable gas, the sensor’s conductivity increases depending on the gas concentration in the air. The TGS 2620 is highly sensitive to the vapors of organic solvents and other volatile vapors, making it suitable for organic vapor detectors/alarms. The complete list of VOCs TGS 2620 senses includes methane, carbon monoxide, iso-butane, hydrogen, and ethanol. Due to the sensing chip’s miniaturization, TGS 2620 requires a heater current of only 42mA and the device is housed in a standard TO-5 package [31].
8. MQ 138: The sensor measures the change in conductivity of a tin dioxide SnO${}_{2}$ semiconductor when exposed to VOCs. In clean air, SnO${}_{2}$ has low conductivity. However, when VOCs are present, they react with the SnO${}_{2}$ and increase its conductivity. The change in conductivity can be measured as a voltage change, which can then be used to determine the concentration of VOCs in the air. The MQ138 sensor is sensitive to various VOCs, including formaldehyde, benzene, toluene, and acetone. It has a working range of 1 to 100 ppm for benzene [32].
9. DHT 22: DHT22 is a commonly used temperature and humidity sensor. The sensor has a dedicated NTC thermistor to measure temperature and an 8-bit microcontroller to output temperature and humidity values as serial data. The sensor can measure temperature from −40 °C to 80 °C and humidity from 0% to 100% with an accuracy of ±1 °C and ±1% [33].

Table 1 provides a summary of the sensors that we used, the VOCs they are sensitive to, and their sensitivity range in parts per million (ppm).

### 3.4. Methodology

**Figure 2:**
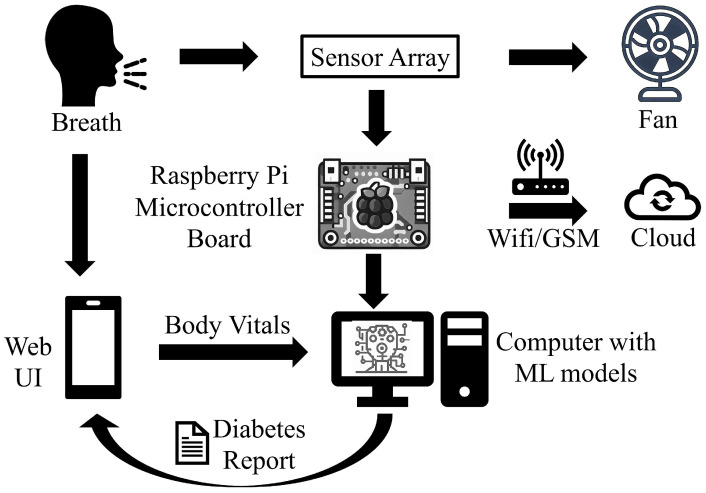
Block diagram of our diabetes detection system.

**Figure 3:**
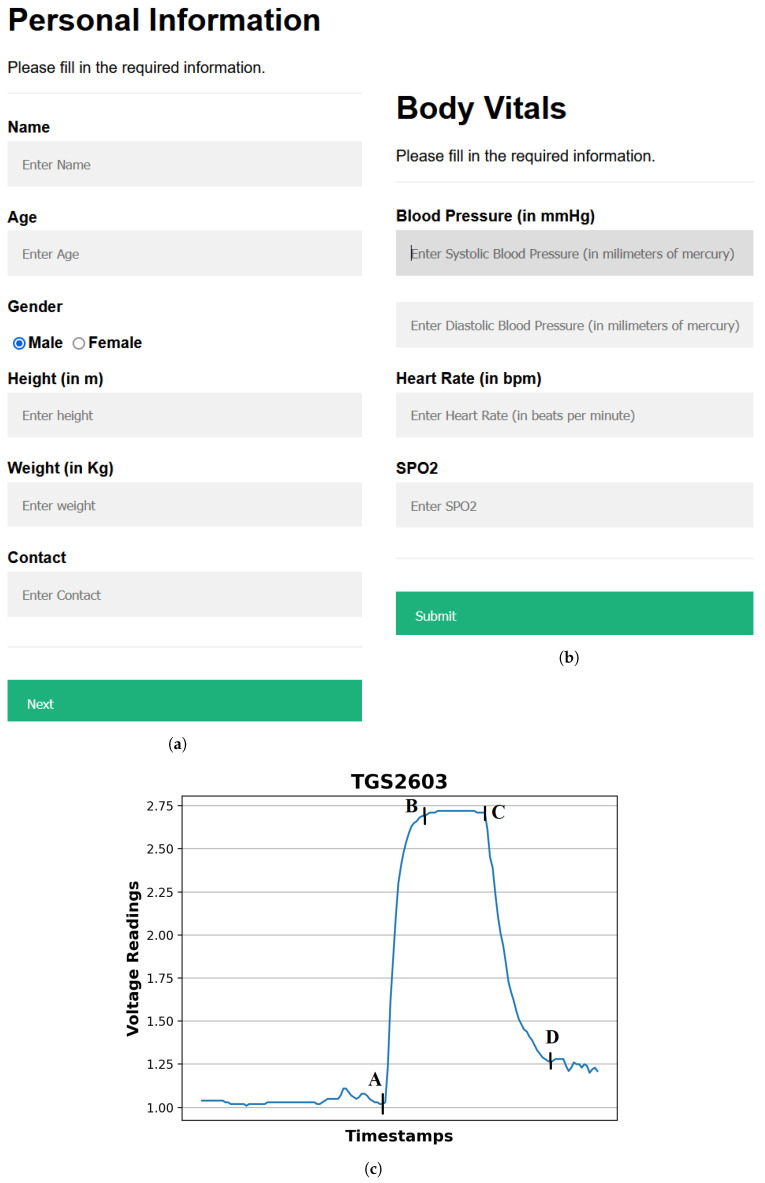
Web interface for entering details and sensor’s response to a breath sample. (**a**) Personal Details Interface. (**b**) Body Vitals Interface. (**c**) TGS2603 Sensor's voltage response to an input breath sample. Segments AB and CD represent the sensor's switching state; Segment BC represents the sensor's stable ON state.

Figure 2 illustrates the methodology behind the breath analysis process performed using our device and can be described as follows:

1. Providing input details using a web-based interface: The procedure starts by entering the user’s demographic and body vitals information using a web-based interface (Figure 3a,b). The demographics include name, age, gender, height, and weight. To record the body vitals of a user, we make them sit in a stable position, rest for five minutes (to make their vitals stable if they have performed some physical activity), and then record their blood pressure, heart rate, and blood oxygen level using standard digital health devices available in the market. These measures can also be self-recorded by a user using digital health devices or a smartwatch and can be entered into the web-based interface. Note: the age recorded in the dataset is when they were detected with diabetes. Furthermore, since most of the T2DM cases occur at ages above 25 years, we have currently collected data from patients with an age of more than 25 years [34].
2. Calibrating the sensors: To ensure accurate sensor readings, we calibrate the sensors to establish stable baselines by validating their readings under reference conditions using fresh air. This implies that the sensors were exposed to a known concentration of VOCs and their outputs were recorded. These data can then be used to create a calibration that can be used to correct the sensor readings for any variation. To obtain a stable baseline from the sensor output, the sensors were preheated with a microheater of the gas sensor. Once the sensor’s output stabilizes under fresh-air conditions, breath sample signatures are obtained from the sensor array (details of sensors’ calibration experiments are described in Section 3.5).
3. Preheat the sensors: The sensors’ temperature increases to a relatively stable level during use, resulting in a change in the baseline response of the sensors. Therefore, the device was switched on for approximately 20 min until the baseline response shown on the host computer was stable.
4. Regular weekly calibration of the sensors: In addition to the initial calibration, we also performed regular calibrations every two weeks to reduce the time drift. This is carried out by exposing the sensors to a set of 10 healthy or non-diabetic breath samples and 3 diabetic breath samples, and verifying that the sensor voltages’ range differs for diabetic participants in comparison to the non-diabetic participants. The results of the regular calibrations were used to update the calibration curve, which ensures that the sensor readings remain accurate over time.
5. Collecting and infusing the breath sample into the device: A (fasting sugar) breath sample collected in a balloon was infused into the sensor-based setup using a drip pipe mounted on top of the soda cup cap. We placed silica gel packets in a device to absorb moisture (or water) present in breath samples. The drip pipe’s one end was attached to the mouth of the inflated balloon, housing the breath sample. The other end was connected to a soda cup cap containing the embedded sensors.
6. Processing the breath sample and recording the data: Upon interaction with the VOCs present in the breath sample, the sensors showed deflection from their baseline (as showing in Figure 3c). The recorded deflection data conveyed through the MQTT protocol are directed into an InfluxDB time series database. The Grafana visualization dashboard facilitates the visualization of real-time sensor responses. The experimental setup comprised a Raspberry Pi hosting the MQTT server, Grafana, Node-RED, and Influx DB running as a Docker container. The sensor voltage readings acted as those of characteristics as they depended on the concentration of VOCs in the breath sample.
7. Getting the setup ready for the following sample: After a reaction time of two seconds, we removed the cup’s cap and mounted it with a fan assembly to expel the breath sample present in the device. Once the voltage readings were restored to their baselines, we stopped recording these for the present sample. At this stage, the setup is ready to process the next breath sample.

The sensor voltages collected for each breath sample represent the characteristics of how various sensors sense the VOCs’ concentrations present in the breath sample, and thus collectively represent an instantaneous breath profile of a person. The breath profile may change with time (based on metabolic processes going on in the body) or due to the consumption of various food items. While collecting the VOC-sensitive sensor data, we also recorded the sensors’ readings for temperature and humidity and normalized the sensor voltage readings using the temperature and humidity sensor readings to negate their effect (if any). Such a collection of normalized sensor-based voltage data and the body vitals data obtained by processing various breath samples collectively forms our dataset. Such normalized feature set and use of silica packets to absorb moisture during breath sample processing helped to minimize problems like air from the oral cavity and the influence of temperature and humidity.

**Figure 4:**
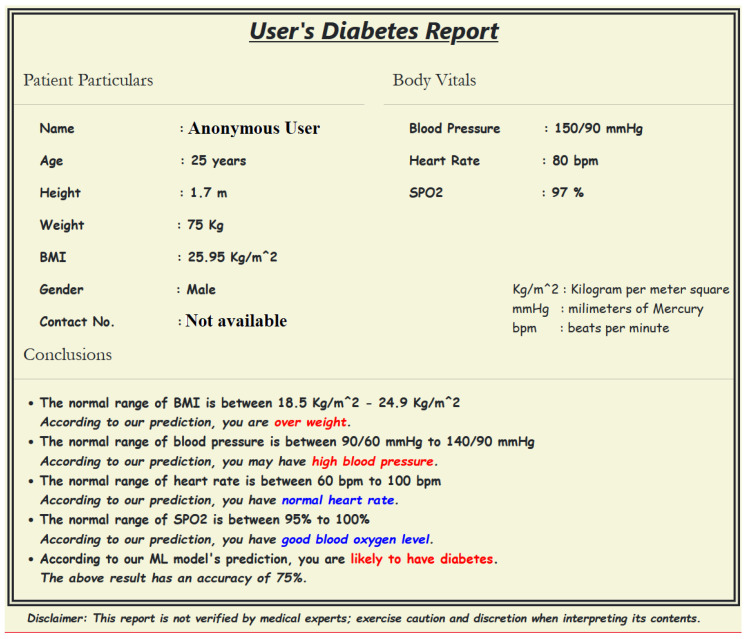
Diabetes report viewing web-interface. Note: The conclusions in blue represent normal readings, while those in red represent the abnormal readings.

We used our dataset to train and validate various ML models and obtain the best-performing ML model, which was used to generate a diabetes prediction report for a new test breath sample, as shown in Figure 4. As shown in Figure 3c, the sensor voltages are irregular before point A and after point D; segment AB represents the switching state where the sensor starts reacting with the VOCs present in the breath; segment BC represents a stable ON state when the sensor voltages become stable; and the segment CD represents the switching phase when the effect of VOCs starts diminishing. The sensors start coming close to their baseline voltages.

DiabeticSense provides instantaneous glucose testing by analyzing the VOCs present in the breath sample at that very instant when submitted for analysis. Also, the user receives an instantaneous response to the test in less than two seconds from our device. Therefore, the testing takes place instantaneously to avoid rapid changes in the concentration of glucose in the blood. Furthermore, we collected five samples from all the patients we considered while developing our ground truth breath-based sensor voltage dataset to ensure accurate results. Similarly, the user may take three to five instantaneous tests using our device (with a gap of three seconds between each test) to confirm the results and minimize the error.

### 3.5. Sensor Calibration Experiments

Since our sensors do not show specificity to specific gases and instead are sensitive to multiple VOCs, we did not have the option to calibrate them using specific gases. However, since our objective is to detect diabetic people whose breath contains an increased concentration of acetone, we performed bi-fold calibration experiments. First, we performed a calibration of our sensors by varying acetone concentrations, i.e., 2, 10, 20, and 40 mL. Specifically, to create a one part per million (ppm) mixture of acetone, we evaporated 2.2302 mL acetone in a 177 m${}^{3}$ dimensions lab and sealed all the openings of the room [14]. Similarly, when we evaporate 4.4604 mL of acetone in the same room, we obtain a 2 ppm acetone mixture. The breath samples collected from this room containing air with a 2 ppm acetone mixture were used for the first set of calibration experiments. The procedure was repeated for calibration with acetone concentrations of 10, 20, and 40 mL. This approach allowed us to observe the voltage changes in the sensor responses at different acetone concentration levels. Next, we tested the voltage deflections using standard breath samples from 10 non-diabetic participants and 3 diabetic participants. Our objective was to check if the voltage displayed for non-diabetic participants remained in the same range and differed diversely from that when checked for diabetic participants.

Further, to address the multi-specificity issues of our sensors, we opted for a cluster of sensors instead of a single sensor to obtain a breath profile of a person and not the specific VOCs. The breath profiles projected by the sensors as voltage defections act as breath representatives of these patients to train our ML models. By employing an array of sensors, we ensured that our ML model had access to a rich set of features, facilitating the discrimination of sensor responses necessary for predicting diabetic and non-diabetic conditions based on the responses obtained from breath samples. Note: the objective of our work is not to discern and detect the concentration of various VOCs but to differentiate between a diabetic person and a non-diabetic person based on their breath profiles (represented by the sensor voltages due to various VOCs).

### 3.6. Clinical Trial Study and Experimental Setting

To evaluate the performance of DiabeticSense, we conducted a clinical trial study by collecting the body vitals and breath samples from 110 patients (36 females and 74 males) in a controlled environment at a reputed national hospital. To collect the breath samples of 110 patients, we asked the patients to blow birthday balloons and record their body vitals using standard digital health devices. To establish and develop the ground truth in data (whether a patient was diabetic), we collected the blood glucose readings from patients’ clinical blood reports from the hospital from which we collected the breath samples.

From the collected breath samples, 100 out of 110 breath samples were finalized after data cleaning and preprocessing. The rejected ten samples had some sensor values missing due to lag in reaching the InfluxDB cloud interface. Of these 100 breath samples, 62 were diabetic and 38 were non-diabetic. We deflated the breath sample balloons one by one into our device to obtain the sensor voltages, and then extracted features from the characteristic curve obtained for the sensor voltages of a sample (Figure 3c). The details of breath sample processing, feature extraction, and ML model development are described in this section. The performance evaluation of the ML models developed using our dataset is shown in Section 4.

### 3.7. Preprocessing the Sensor Voltages

Preprocessing the sensor voltages is performed to remove noisy voltage points and select the relevant ones for feature extraction and ML model development. As Figure 3c shows, the sensor voltages at the onset and end of the sample processing, i.e., at points A and D, are irregular (thus are noise) and must be removed. Therefore, for feature extraction and ML model development, for each sensor, we sorted the sensor voltages obtained for each breath sample in descending order and took the top 5 sensor voltage readings, which acted as the true representatives of the breath sample analysis between points A and D. The objective here is to capture the sensor voltages for on state represented by the segment AB in Figure 3c. The dataset formed after obtaining the body vitals information and recording the sensor voltages for breath samples collected from 100 finalized breath samples is available at https://zenodo.org/record/8274426 (accessed on 23 September 2023). Note: we did not use the sensor voltages directly for ML model training; instead, the features extracted from these sensor voltages and the body vitals data were collectively provided as input for ML model training and testing. Also, we considered the top 5 sensor voltages for each breath sample obtained from all sensors.

### 3.8. Feature Extraction

For each of the breath samples and its top 5 sensor voltages, we extracted various spatial and frequency features such as curve magnitude, slope, first and second derivatives, etc., as mentioned in [4]. The complete list of features used is listed in Table 2. The features extracted for each breath sample were concatenated as a single feature vector. The complete set of feature vectors obtained by feature extraction from breath samples forms our feature matrix for ML model development. To balance our feature set, we experimented with SMOTE (https://imbalanced-learn.org/stable/references/generated/imblearn.over_sampling.SMOTE.html (accessed on 23 September 2023)) and ADASYN (https://imbalanced-learn.org/stable/references/generated/imblearn.over_sampling.ADASYN.html (accessed on 23 September 2023)) techniques, with ADASYN generating the best performance results (discussed in Section 4). Further, we scaled our feature set using the MinMaxScalar (https://scikit-learn.org/stable/modules/generated/sklearn.preprocessing.MinMaxScaler.html (accessed on 23 September 2023)) function of Scikit learn (https://scikit-learn.org/ (accessed on 23 September 2023)) to remove the bias toward individual features.

### 3.9. ML Model Development

To perform the diabetes prediction task, we experimented with the following ten state-of-the-art ML algorithms by training and testing the feature matrix obtained by processing the breath samples, as discussed in the previous subsection. A random 80:20 split of the feature set was performed, whereby 80% of the feature set was deployed for training and 5-fold cross-validation, while the remaining 20% of the feature set was used for testing.

1. Gradient Boosting (G-Boost): G-Boost creates a stage-wise model and generalises the model by allowing for the optimization of an arbitrary differentiable loss function. Gradient boosting combines weak learners into a single strong learner in an iterative fashion. As each weak learner is added, a new model is fitted to provide a more accurate estimate of the response variable [52,53].
2. Decision Tree (DT): A DT is developed by recursively splitting data based on feature values to develop subsets that are as pure as feasible, which means that each subset mainly comprises instances of a single class [54].
3. K-Nearest Neighbours (KNNs): KNNs do not make any underlying assumptions about data distribution. Given some prior data (training data), KNNs classify coordinates identified by an attribute [54].
4. Ridge: Ridge regression enhances regular linear regression by slightly changing its cost function, which results in less overfit models [55].
5. Lasso: Lasso is a regression analysis method that performs both variable selection and regularization to enhance the prediction accuracy and interpretability of the resulting statistical model. For lasso, the coefficient estimates do not need to be unique if covariates are collinear. Lasso’s ability to perform subset selection relies on the form of the constraint and has a variety of interpretations, including in terms of geometry, Bayesian statistics, and convex analysis [56,57].
6. Elastic Net (ENet): ENet combines the two most popular regularized variants of linear regression: ridge and lasso. Ridge utilises an L2 penalty, and lasso uses an L1 penalty. ENet uses both the L2 and the L1 penalty [58].
7. Logistic Regression: It is used for predicting the categorical dependent variable using a given set of independent variables. Logistic regression predicts the output of a categorical dependent variable. Therefore the outcome must be a categorical or discrete value. It can be either yes or no, 0 or 1, true or false, etc., but instead of giving the exact value as 0 and 1, it gives the probabilistic values between 0 and 1 [59].
8. Support Vector Machines (SVMs): SVMs operate by determining the appropriate hyperplane for separating various classes in the data space. The hyperplane is chosen to maximize the margin, which is the distance between the hyperplane and the nearest data points of each class, also known as support vectors [60].
9. eXtreme Gradient Boosting (XG-Boost): XG-Boost is an open-source software library with a regularising gradient boosting framework for C++, Java, Python, R, Julia, Perl, and Scala. It works on Linux, Windows, and macOS. From the project description, it aims to provide a scalable, portable and distributed gradient boosting (GBM, GBRT, GBDT) library. It runs on a single machine, and the distributed processing frameworks Apache Hadoop, Apache Spark, Apache Flink, and Dask [12].
10. Random Forest (RF): The RF algorithm generates multiple DTs during training by selecting random subsets of the original dataset and random subsets of characteristics for each tree. Each DT in the RF is developed using a technique known as recursive partitioning, which involves repeatedly splitting the data into subsets depending on the most discriminatory attributes, resulting in a tree-like structure [60].

Each ML model built using these techniques acts as a binary classifier. For instance, when testing for diabetes with a particular breath sample as input, an ML model marks it as *likely to be diabetic* or *likely to be non-diabetic*. To obtain the best-performing ML models, we performed hyper-tuning of these ML classifiers using various parameter combinations listed in Table 3.

Also, to ensure the reliability of our results, we performed k-fold cross-validation. Higher values of k limit the number of data points in a validation set, and a lower value would increase the risk of bias in the dataset. We therefore selected a threshold value of k = 5 for our experiments.

### 3.10. Evaluation Metrics

To obtain the best-performing ML model for our diabetes prediction device, we compared the performance of various ML models trained on our dataset using the evaluation metrics described as follows: We selected *Accuracy* (https://scikit-learn.org/stable/modules/generated/sklearn.metrics.accuracy_score.html (accessed on 23 September 2023)), *F1 Score* (http://scikit-learn.org/stable/modules/generated/sklearn.metrics.f1_score.html (accessed on 23 September 2023)) and *ROC curve area* (http://scikit-learn.org/stable/auto_examples/model_selection/plot_roc.html (accessed on 23 September 2023)) metrics as the evaluation metrics of our work, defined as follows:

(1) $$\begin{array}{c} Accuracy=\frac{Number\;of\;correct\;predictions}{Total\;number\;of\;predictions} \end{array}$$

(2) $$\begin{array}{c} F1\;Score=\frac{2\times Precision\times Recall}{Precision+Recall} \end{array}$$

where

(3) $$\begin{array}{c} Precision=\frac{true\;positive}{true\;positive+false\;positive} \end{array}$$

and

(4) $$\begin{array}{c} Recall=\frac{true\;positive}{true\;positive+false\;negative} \end{array}$$

Because the *F*1 score captures both the effect of *Precision* and *Recall*, we computed the *F1 Score* values and their respective standard deviation values (or error values) only. The higher the *F1 Score*, the better the model’s prediction accuracy.

*ROC Area Under the Curve (AUC) metric* evaluates the output quality. An ROC curve is a plot that features the *true positive rate*. (marked as the Y-axis) versus the *false positive rate* (marked as the X-axis) for an experiment. The point at the top-left corner of the plot depicts the point of the most ‘ideal’ behavior having the {*false positive rate, true positive rate*} pair value as {0, 1}. Thus, a larger area under the curve signifies a better quality output. We therefore selected the ROC curve area as our second evaluation metric.

Since the highest *Accuracy* value, *F1 Score* value, and *ROC AUC* value may differ across the models, we took the average of these metrics as the final accuracy measure of a model (*MeanAcc* defined in Equation (5)).

### 3.11. Selecting the Best-Performing Model for Diabetes Prediction

Since we used multiple evaluation metrics to compare the performance of these ML models, we selected the best-performing model by taking an average (or mean) of the performance metrics for each of the ML models, described in detail as follows:

We define the best-performing ML model as the one with the highest *MeanAcc* measure value, where *MeanAcc* is represented by Equation (5). There, *A* represents the set of ML algorithms and Π is the tuning parameter combinations set, such that $\Delta^{\alpha ,\pi }$ represents the ML model built using the ML algorithm α with the parameter combination π, such that α∈A,andπ∈Π. The complete list of ML algorithms (*A*) and the hyper-tuning parameters used (Π) is found in Table 3.

(5) $$MeanAcc\left(\Delta^{\alpha ,\pi }\right)=\left(F1\;Score\left(\Delta^{\alpha ,\pi }\right)+Accuracy\left(\Delta^{\alpha ,\pi }\right)+ROC\;AUC\left(\Delta^{\alpha ,\pi }\right)\right)/3$$

As shown in Equation (5) above, *MeanAcc* is computed simply by performing an average of *F*1 *Score, Accuracy*, and *ROC AUC* metrics values obtained by using the best hyper-tuned parameter values. We have defined these evaluation metrics in Section 3.10. Thus, the problem of finding the best-performing model trained on our dataset (containing sensor and body vital readings) to perform the diabetes prediction for a test breath sample can be defined as follows:

(6) $$\max_{\alpha ,\pi }^{A,\Pi }MeanAcc\left(\Delta^{\alpha ,\pi }\right),\;where\;\alpha \in A,\pi \in \Pi$$

As represented by Equation (6), the best-performing model is defined as the model yielding the highest *MeanAcc* when trained using the optimal parameters obtained using the hyper-tuning procedure. The hyper-tuning procedure was run for each of the ML algorithms using 20,000 iterations and the parameter combinations listed in Table 3.

### 3.12. Ethical Consideration

The study has received ethical approval from the institutional review board. Informed consent was obtained from all participants before their involvement.

## 4. Results

**Figure 5:**
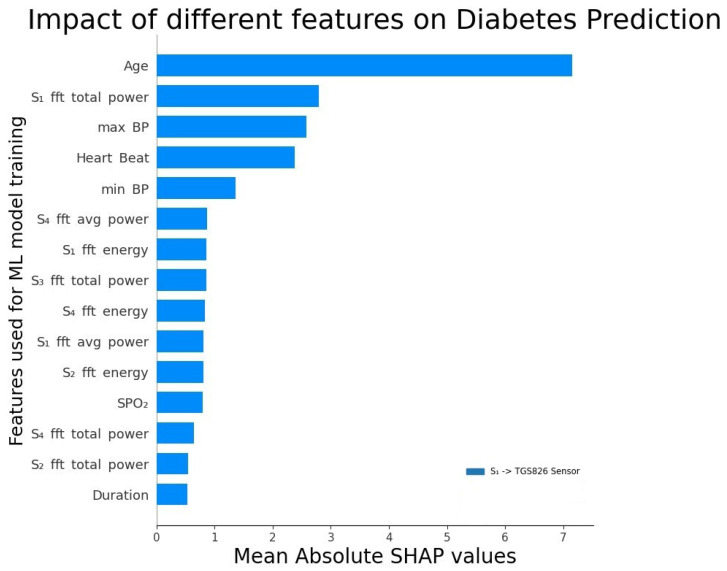
SHAP plot representing the impact of features on classification decision.

We performed various ML model training experiments with the complete feature set listed in Table 2, and obtained a reduced feature set in Table 4 that resulted in the best performance results (i.e., yielding the highest *MeanAcc* given in Equation (5)). Figure 5 shows the Shapley additive explanations (SHAPs) plot (https://shap.readthedocs.io/en/latest/ (accessed on 23 September 2023)) of a subset of features used in our dataset, showing the impact of features on the classification decision, i.e., diabetic or non-diabetic predicted by an ML model. The features shown in this plot are the most positively contributing features toward the classification decision of predicting if a person is diabetic or not.

As discussed in Section 3.9, we split our feature set into a training and testing set using an 80:20 ratio, where 80% of the records are used in training (with hyper-tuning) and k-fold cross-validation (k = 5), and the remaining 20% are used for testing with the best hyper-tuned ML models. Table 5a shows the five-fold cross-validation results for comparing various ML models (using their hyper-tuned parameter values listed in Table 6 and reduced features (Table 4) based on the evaluation metrics described in Section 3.10.

As our dataset contained features obtained from breath samples of 62 diabetic and 38 non-diabetic participants, we experimented using SMOTE and ADASYN techniques for balancing the feature set (both training and test splits). Table 5a shows the performance improvement obtained by balancing the feature set using SMOTE and ADASYN techniques, with the ADASYN-balanced Gradient Boost ML model yielding the best results. SMOTE has also been used previously to balance feature sets before performing ML model training [57]. Table 5b shows the test result performance evaluation metrics values obtained by using the ADASYN-balanced feature set and hyper-tuned k-fold models of the considered ML algorithms on the 20% feature set.

## 5. Discussion

### 5.1. Performance Evaluation

As shown in Table 5a, the gradient boosting (G-Boost) algorithm performs the best when an ADASYN balanced feature is used, with a mean accuracy of 86.6%. The XG-Boost algorithm has been proven to perform best in detecting acetone concentrations in breath [12]. However, our dataset comprises sensor voltage readings for different VOCs and is not limited to acetone. We experimented with various state-of-the-art ML algorithms, including gradient Boosting and XG-Boost algorithms, with G-Boost performing the best in our case. In addition, because this work was performed by training XG-Boost on simulation-based data with different acetone concentrations and not real-time breath-based data, we could not directly compare [12]’s accuracy with ours. Some of the reasons for G-Boost performing the best in our case could be its capability to (a) capture intrinsic relationships between body vital features and sensor-based features, (b) capture feature importance scores and apply them in prediction, (c) combine multiple weak learners to form a robust predictive model, (d) capture and handle nonlinearity, (e) be robust to outliers, (f) have ensemble diversity, (g) prevent overfitting and enhance generalization using regularization, and (h) handle the missing data and reduce the need for extensive data preprocessing.

### 5.2. Feature Analysis

Figure 5 shows the SHAP plot obtained for our feature set using the best-performing model; that is, Gradient Boost with optimally hyper-tuned parameters. SHAP plots are a visualization technique used in ML to identify the features contributing the most individuals for the prediction task. It is interesting to note from the SHAP plot that the body’s vital features such as age, BP, heartbeat, SPO${}_{2}$, and most of the FFT features contribute the most to the classification process of diabetes detection. Furthermore, for sensor voltage, the main contributors to the diabetes prediction process were TGS826, TGS2603, TGS2610, and TGS2620, which validates the observations from the results in [4]. However, our work had different best-contributing features, as shown in Figure 5. The complete set of features used listed in Table 4, instead of the wavelet and magnitude features, which are listed in the literature [4]. These sensors are sensitive to VOCs such as ammonia, LP gas, propane, butane, alcohol, organic solvent vapors, amine series, and sulfurous gasses.

### 5.3. Performance Comparison with the Existing Works

Recently, the authors of [12] reported a similar diabetes detection work by applying the XG-Boost model on a breath-simulated dataset comprising sensor voltages obtained at different acetone concentrations. However, this study focused only on acetone and did not consider other VOCs present in the breath and their contributions to diabetes. Furthermore, because the accuracy measures were computed on a simulated dataset, it was not possible to compare the work directly with this study, where the models were on features extracted from the real-time clinical breath-based data. Furthermore, this study considered various other VOCs beyond acetone (see Table 1 of Section 3).

Another exciting work [13] on real-time breath-based data makes use of the support vector ordinal regression technique to classify a breath sample into four ordinal groups, i.e., well controlled, somewhat controlled, poorly controlled, and not controlled, with an accuracy of 68.66% [13]. Our work differs from [13] in terms of the type of sensors used and the approach used; that is, the classification of real-time data versus the probabilistic approach. Furthermore, the current study reports an accuracy improvement of 20.72% over this work. Finally, the authors in [24] have predicted the loss of protective sensitivity in the foot in over 111 patients diagnosed with diabetes mellitus. The study reported a true positive rate of 81% and a false positive rate of 95.5%. Our work differs from [24] in terms of the focus, where we classify patients as diabetic or not, whereas [24] classifies patients as losing protective sensitivity in the foot.

We could not find any other work that performed a similar ML classification on real-time breath samples collected from patients with diabetes. Furthermore, using body vitals data from our feature set in addition to sensor-based features is one of the major novelties of our study.

## 6. Conclusions, Limitations, and Future Work

T2DM, a prevalent chronic metabolic disorder, requires the continuous monitoring of blood glucose levels. With 95% of T2DM cases reported worldwide and 46.1% reported as undiagnosed (or diagnosed late) by IDF and WHO, a need exists for novel non-invasive pre-diagnostic methods that facilitate early detection and control. To counter the above problem, we propose a non-invasive multi-sensor Internet of Things (IoT)-based diabetes detection system (DiabeticSense) that, given a patient’s breath sample and body vitals, generates an ML prediction of whether the patient is likely to have diabetes. Our multi-sensor system comprises various MOS-type sensors sensitive to various VOCs, which help to differentiate the breath samples of a diabetic patient from those of a non-diabetic patient. After processing 100+ breath samples using our device, and training various ML models on the sensor data collected, we conclude that the gradient boosting algorithm yields the highest accuracy of 86.6%. However, an immense scope exists to improve the accuracy by performing various feature engineering techniques, improving the sample size, and including more sensors to support the decision. Currently, DiabeticSense is only capable of detecting whether a person is diabetic or not and cannot predict the exact blood sugar levels of a person. However, we are working on adding an instantaneous blood sugar monitoring capability into DiabeticSense by increasing the feature set size and thus improving the ML model predictions’ accuracy for this task.

We plan to add more VOC-sensitive sensors to our device, such as TGS-1820 and MQ-3, as reported in the literature, contributing positively toward diabetes detection by contributing to acetone detection. Though our current diabetes detection system provides a low-cost and portable solution, we are continuously improving our system design to make it portable and compact in nature to facilitate ease of use. We are also working on moving the entire computation to the cloud, thereby providing access to devices using a light mobile interface. We plan to conduct an ablation study to determine the effect of working with a reduced set of sensors on the prediction. Our diabetes detection device can be easily implanted in areas devoid of proper medical conditions in rural and remote locations of our country. Our device can act as an excellent pre-diagnostic tool for diabetes detection and the early detection of diabetes and its treatment. We are also working on improving the design of our device and making it capable of instantaneous blood sugar monitoring using breath. Once we successfully reduce the size of our device and make it more compact, it could easily be used as a handy device for diabetes detection and blood sugar monitoring without the inconveniences of prick-testing. Finally, we plan to extend our diabetes detection breath analysis approach for heart disease prediction using the VOCs associated with breath biomarkers of heart patients.

Our diabetes detection device can be easily implanted in areas devoid of proper medical conditions, such as rural and remote locations of our country. Our device can act as an excellent pre-diagnostic tool for diabetes detection and help in the early detection of diabetes and its treatment. We are also working on improving the design of our device and making it capable of instantaneous blood sugar monitoring using breath. Once we successfully reduce the size of our device and make it more compact, it can easily be used as a handy device for diabetes detection and blood sugar monitoring without the inconvenience of prick-testing.

## Figures and Tables

**Table 1:** MOS-type electrochemical sensors used in the device.

Sensor Model	VOCs Sensitivity	Sensitivity Range (in ppm) ^1^
TGS 826	iso-butane, ethanol, ammonia, and hydrogen	30–5000
TGS 2610	ethanol, hydrogen, methane, iso-butane, and propane	500–10,000
TGS 822	methane, carbon monoxide, iso-butane, n-hexane, benzene, ethanol, and acetone	50–5000
TGS 2602	hydrogen, hydrogen sulfide, ammonia, ethanol, and toluene	1–30
TGS 2600	methane, carbon monoxide, iso-butane, ethanol, and hydrogen	1–100
TGS 2603	hydrogen sulfide, hydrogen, methyl mercaptan, trimethyl amine, and ethanol	1–10
TGS 2620	methane, carbon monoxide, iso-butane, hydrogen, and ethanol	50–5000
MQ 138	formaldehyde, benzene, toluene, and acetone	5–500
DHT 22	Humidity (H) and Temperature (T)	H: 0–100 RH, T: −40–80 °C

^1^ ppm: parts per million.

**Table 2:** List of features extracted from sensor voltage data.

Base Feature	Feature Used	Description
CurveMagnitude	abs(CurveMagnitude) [35]	The absolute value of curve magnitude values.
	max(CurveMagnitude) [36]	The maximum of curve magnitude values.
	min(CurveMagnitude) [37]	The minimum of curve magnitude values.
	mean(CurveMagnitude) [38]	The mean or average of curve magnitude values.
	stdDev(CurveMagnitude) [39]	The median curve magnitude values.
FirstDerivative [40]	max(FirstDerivative)	The maximum of first derivative of signal values.
	min(FirstDerivative)	The minimum of first derivative of signal values.
	mean(FirstDerivative)	The mean of first derivative of signal values.
	abs(FirstDerivative)	The absolute value of the first derivative.
	stdDev(FirstDerivative)	The square root of the variance of the first derivative.
SecondDerivative [40]	max(SecondDerivative)	The maximum of second derivative of signal values.
	min(SecondDerivative)	The minimum of second derivative of signal values.
	mean(SecondDerivative)	The mean of second derivative of signal values.
	abs(SecondDerivative)	The absolute value of the second derivative.
	stdDev(SecondDerivative)	The square root of the variance of the second derivative.
Slope and Integral of five intervals [41]	Slope of five intervals	The slope of the five intervals of the curve ^1^.
	Integral of five intervals	The integral of the five intervals of the curve ^1^.
Phase	$\int_{M(t_{i})}^{M(t_{\left(i+1\right)})}D\;dM$	It represents the integral of derivative over the magnitude values [42].
Fast Fourier Transform (fft) [43,44]	phase	The phase is calculated based on the fft of the sensors’ response.
	powerSpectrum	The square of the absolute value of fft transform.
	spectralEntropy	It represents the entropy of power spectrum.
Wavelet [45]	waveletCoeffs	Coefficients of wavelet transformation of the sensor’s response signal.
Peak [46]	height	The height of the peak.
	width	The width of the peak.
	area	The trapezoidal area of the peak.
Shape	skewness [47]	The measure of the asymmetry of a distribution, where a positive skew indicates a longer tail on the right side and a negative skew indicates a longer tail on the left side.
	kurtosis [48]	The measure of the tailedness of a distribution; a positive value indicates fatter tails and a negative value indicates thinner tails.
	entropy [49]	The measure of the disorder or randomness of a shape; a higher entropy indicates a more disordered or random shape.
Auto-Regressive (AR) [50]	coefficients	These represent the relationships between past and current values of the model.
	predictionError	The difference between the actual observed value and the AR model’s predicted value.
Short-time Fourier transform (STFT) [51]	dominantFrequency	The frequency component that has the highest magnitude of the signal.
	avg(magnitude(STFTcoeffs))	The average magnitude of the STFT coefficients, calculated by taking the mean of the magnitudes over all the time frames.
	Sum(magnitude(STFTcoeffs))	The sum of the magnitudes of all the STFT coefficients.
	energy(STFT)	The overall power of the signal in the frequency domain.
	centroid(STFTcoeffs)	The weighted average of the frequencies in the STFT, where the weights are the magnitudes of the STFT coefficients.
	bandwidth(STFT)	The range of frequencies represented by a single STFT coefficient, determined by the window length.
	rolloff(STFT)	The frequency at which the magnitude of the STFT coefficients drops to −3dB, typically used as a measure of the sharpness of the transition between the passband and the stopband.

^1^ Five equal distance intervals are created from the sensor’s response voltages for a breath sample.

**Table 3:** Parameter combination of different ML techniques used for diabetes prediction.

ML Classifiers	Parameter Name	Parameter Values
Decision Tree	criterion	(‘gini’, ‘entropy’, ‘log_loss’)
	splitter	(‘best’, ‘random’)
	max depth	(2 to 10, step size of 1)
	min samples split	(2 to 10, step size of 1)
Support Vector	C	(0.1 to 10, step size of 0.1)
	kernel	(‘linear’, ‘poly’, ‘rbf’, ‘sigmoid’, ‘precomputed’)
	degree	(3 to 10, step size of 1)
	gamma	(‘scale’, ‘auto’, ‘float’) with (0.001 to 1, step size of 0.005) for ‘float’
Gradient Boost	learning rate	(0.01 to 10, step size of 0.01)
	n estimators	(5 to 500, step size of 5)
	subsample	(0.01 to 1, step size of 0.01)
	criterion	(‘friedman mse’, ‘squared error’)
	min samples split	(2 to 10, step size of 1)
	max depth	(2 to 10, step size of 1)
Random Forest	n estimators	(5 to 500, step size of 5)
	criterion	(‘gini’, ‘entropy’, ‘log loss’)
	min samples split	(2 to 10, step size of 1)
	max depth	(2 to 10, step size of 1)
	max features	(‘sqrt’, ‘log2’)
	min samples leaf	(1 to 10, step size of 1)
K-NNeighbors	n neighbors	(5 to 100, step size of 5)
	weights	(‘uniform’, ‘distance’)
	algorithm	(‘auto’, ‘ball tree’, ‘kd tree’, ‘brute’)
	leaf size	(30 to 100, step size of 3)
Elastic Net	alpha	(0.01 to 1, step size of 0.01)
	l1 ratio	(0.01 to 1, step size of 0.01)
	fit intercept	(True, False)
	max iter	(1000 to 5000, step size of 100)
	selection	(‘cyclic’, ‘random’)
Ridge	solver	(‘auto’, ‘svd’, ‘cholesky’, ‘lsqr’, ‘sparse_cg’, ‘sag’)
	fit intercept	(True, False)
	max iter	(1000 to 5000, step size of 100)
Lasso	alpha	(0.1 to 10, step size of 0.1)
	fit intercept	(True, False)
	copy X	(True, False)
	max iter	(1000 to 5000, step size of 100)
	selection	(‘cyclic’, ‘random’)
Logistic Regression	penalty	(‘l1’, ‘l2’, ‘Elastic Net’, None)
	dual	(True, False)
	C	(0.1 to 10, step size of 0.1)
	fit intercept	(True, False)
	solver	(‘lbfgs’, ‘liblinear’, ‘newton-cg’, ‘newton-cholesky’, ‘saga’, ‘sag’)
	max iter	(1000 to 5000, step size of 100)
	multi class	(‘auto’, ‘ovr’, ‘multinomial’)
XG-Boost	max depth	(1 to 10, step size of 1)
	alpha	(0.1 to 10, step size of 0.1)
	booster	(‘gbtree’, ‘gblinear’)
	eta	(0.01 to 1, step size of 0.01)
	min child weight	(1 to 10, step size of 1)

**Table 4:** Features used for experiments (subset of features described in Table 2).

Feature	Description
Age	Age of the user
Gender	Gender of the user, i.e., male, female, or other
BP	User’s max and min BP values
SPO${}_{2}$	Oxygen level in blood
Heart Rate	Heart rate of the patient
Fast Fourier Transform (fft)	phase
	powerSpectrum
	spectralEntropy
Phase	$\int_{M(t_{i})}^{M(t_{\left(i+1\right)})}D\;dM$
FirstDerivative	max(FirstDerivative)
	min(FirstDerivative)
	mean(FirstDerivative)
	abs(FirstDerivative)
	stdDev(FirstDerivative)
SecondDerivative	max(SecondDerivative)
	min(SecondDerivative)
	mean(SecondDerivative)
	abs(SecondDerivative)
	stdDev(SecondDerivative)
Slope and Integral of five intervals	Slope of five intervals ^1^.
	Integral of five intervals ^1^.

^1^ Five equal distance intervals are created from the sensor’s response voltages for a breath sample.

**Table 5:** Performance comparison using five-fold cross-validation, ADASYN and SMOTE balancing, and 80:20 split for training and testing with hyper-tuned ML models.

(a) Five-Fold Cross Validation Performance Metrics Scores of Various ML Classifiers					
**ML Classifiers**	**BalancingTech**	**MeanAccuracy**	**MeanF1Score**	**MeanROC**	**MeanAcc ^1^**
Decision Tree	ADASYN	0.847	0.843	0.844	0.845
	SMOTE	0.796	0.793	0.801	0.797
	UnBalanced	0.713	0.662	0.666	0.68
Support Vector	ADASYN	0.695	0.69	0.693	0.693
	SMOTE	0.717	0.706	0.715	0.713
	UnBalanced	0.625	0.382	0.492	0.5
Gradient Boost	ADASYN	0.866	0.865	0.868	0.866
	SMOTE	0.816	0.812	0.819	0.816
	UnBalanced	0.763	0.741	0.774	0.759
Random Forest	ADASYN	0.817	0.813	0.815	0.815
	SMOTE	0.737	0.73	0.753	0.74
	UnBalanced	0.663	0.582	0.591	0.612
K-NNeighbors	ADASYN	0.601	0.597	0.604	0.601
	SMOTE	0.668	0.656	0.664	0.663
	UnBalanced	0.575	0.484	0.548	0.536
Elastic Net	ADASYN	0.726	0.684	0.749	0.72
	SMOTE	0.775	0.763	0.77	0.769
	UnBalanced	0.688	0.634	0.648	0.657
Ridge	ADASYN	0.754	0.746	0.762	0.754
	SMOTE	0.814	0.807	0.809	0.81
	UnBalanced	0.725	0.679	0.69	0.698
Lasso	ADASYN	0.785	0.732	0.805	0.774
	SMOTE	0.755	0.745	0.754	0.751
	UnBalanced	0.7	0.637	0.652	0.663
Logistic Regression	ADASYN	0.683	0.669	0.693	0.687
	SMOTE	0.824	0.816	0.817	0.819
	UnBalanced	0.713	0.66	0.668	0.68
XG-Boost	ADASYN	0.867	0.857	0.864	0.86.3
	SMOTE	0.815	0.808	0.81	0.811
	UnBalanced	0.688	0.626	0.646	0.653
**(b) Test Performance Metrics Scores of various ML Classifiers**					
**ML Classifiers**		**Accuracy**	**F1 Score**	**ROC Area**	**MeanAcc ^1^**
Decision Tree		0.65	0.601	0.621	0.624
Support Vector		0.55	0.54	0.54	0.543
Gradient Boost		0.85	0.84	0.833	0.841
Random Forest		0.55	0.436	0.51	0.499
K-NNeighbors		0.45	0.449	0.449	0.449
Elastic Net		0.5	0.479	0.485	0.488
Ridge		0.6	0.56	0.576	0.579
Lasso		0.45	0.437	0.439	0.442
Logistic Regression		0.5	0.479	0.485	0.488
XG-Boost		0.75	0.733	0.732	0.738

^1^ Computed using Equation (5).

**Table 6:** Best hyper-tuned parameter values for the ML techniques.

ML Classifiers	Hyper Tuned Parameter Values
Decision Tree	criterion: ‘entropy’, splitter: ‘best’, max depth: 5, min samples split: 2
Support Vector	C: 10, kernel: ‘rbf’, degree: not relevant ^1^, gamma: ‘auto’
Gradient Boost	learning rate: 1, n estimators: 100, subsample: 1, criterion: ‘friedman mse’, min samples split: 2, max depth: 3
Random Forest	n estimators: 100, criterion: ‘entropy’, min samples split: 2, max depth: 9, max features: ‘sqrt’, min samples leaf: 1
K-NNeighbors	n neighbors: 7, weights: ‘distance’, algorithm: ‘auto’, leaf size: 30
Elastic Net	alpha: 0.1, l1 ratio: 0.5, fit intercept: ‘True’, max iter: 1000, selection: ‘cyclic’
Ridge	solver: ‘auto’, fit intercept: ‘True’, max iter: 1000
Lasso	alpha: 0.1, fit intercept: ‘True’, copy X: ‘True’, max iter: 1000, selection: ‘cyclic’
Logistic Regression	penalty: ‘l2’, dual: ‘False’, C: 10, fit intercept: ‘True’, solver: ‘lbfgs’, max iter: 1000, multi class: ‘ovr’
XG-Boost	max depth: 5, alpha: 0.1, booster: ‘gbtree’, eta: 0.3, min child weight: 1

^1^ Only relevant for poly or sigmoid kernel.

## Data Availability

Data available on request due to restrictions, e.g., privacy or ethical. The health care data that we collected may have some privacy considerations concerning patients. Therefore, to ensure the privacy of patients, the data is not publicly available, and is available on request from the corresponding author.

## Abbreviations

The following abbreviations are used in this manuscript:

T2DM	Type 2 diabetes mellitus
IoT	Internet of Things
IDF	International Diabetes Foundation
WHO	World Health Organization
VOC	Volatile Organic Compounds
MOS	Metal-Oxide-Semiconductor
ML	Machine Learning
BGL	Blood glucose level
E-Nose	Electronic Nose
DiabeticSense	Non-invasive multi-sensor diabetes detection device
ADC	Analog-to-digital converter
LP	Liquefied petroleum
ppm	parts per million
mL	milli-Litre
abs	absolute
max	maximum
min	minimum
mean	mean or average
stdDev	standard deviation
avg	average
G-Boost	Gradient Boosting
DT	Decision Tree
KNNs	K-Nearest Neighbours
ENet	Elastic Net
SVMs	Support Vector Machines
XG-Boost	eXtreme Gradient Boosting
RF	Random Forest
AUC	Area Under the Curve
MeanAcc	Mean Accuracy
SHAPs	Shapley additive explanations
BP	Blood Pressure
SPO${}_{2}$	Oxygen level in blood
FFT	Fast Fourier Transform
SMOTE	Synthetic Minority Oversampling TEchnique
MSE	Mean Square Error
sqrt	Square root
T	Temperature
H	Humidity
°C	degree Celsius
